# An integrative pan-cancer analysis of USP37 and functional validation in pancreatic cancer

**DOI:** 10.3389/fcell.2025.1659747

**Published:** 2025-08-25

**Authors:** Jiafei Chen, Liang Lin, Dongxing Chen, Jingui Wang, Wuhan Zhou

**Affiliations:** ^1^ The School of Clinical Medicine, Fujian Medical University, Fuzhou, Fujian, China; ^2^ Department of Hepatobiliary Surgery, The First Hospital of Putian City, Chengxiang, Fujian, China

**Keywords:** USP37, pan-cancer, pancreatic cancer, prognosis, biomarker

## Abstract

**Background:**

USP37, a versatile deubiquitinase, plays a pivotal role in numerous cellular functions. Although its involvement in cancer development is well-established, the comprehensive pan-cancer analysis of USP37 remains relatively uncharted.

**Methods:**

RNA sequencing data from both normal and cancerous tissues were retrieved from The Cancer Genome Atlas (TCGA) and Genotype-Tissue Expression (GTEx) databases. Genomic alterations in USP37 across multiple cancer types were examined using cBioPortal. Protein-related information on USP37 was sourced from the Human Protein Atlas (HPA) and Protein-Protein Interaction (PPI) databases. Additionally, Western blotting was conducted to evaluate USP37 expression in clinical samples and pancreatic cancer cell lines. The prognostic relevance of USP37 across various cancers was analyzed using univariate Cox regression and Kaplan–Meier survival curves. Gene Set Enrichment Analysis (GSEA) was performed to identify cancer hallmarks associated with USP37. USP37 protein levels were quantified via immunoblotting, and *in vitro* and *in vivo* functional assays were employed to assess its role in the proliferation of pancreatic cancer cells.

**Results:**

USP37 was found to be aberrantly expressed in several tumor types, with significant association with poor prognosis in certain cancers, including pancreatic cancer. Its expression was also strongly correlated with immune regulators, tumor mutational burden (TMB), and microsatellite instability (MSI), highlighting its potential as a predictive marker for immunotherapy outcomes. Functional assays demonstrated that USP37 fosters proliferation, migration, and invasion in pancreatic cancer cells, further underscoring its role as an oncogene.

**Conclusion:**

USP37 holds promise as a biomarker and therapeutic target in clinical oncology, providing new insights into its function in cancer.

## 1 Introduction

Cancer poses a significant threat to public health worldwide, with both incidence and mortality rates rising rapidly each year (Bray et al., 2024). Despite considerable efforts to enhance cancer diagnosis and treatment, survival rates remain disheartening, highlighting the need for more effective approaches (Bray et al., 2024; Dyba et al., 2021). Concurrently, the financial burden of cancer has severely impacted healthcare systems and economies across the globe, straining resources and complicating patient care (McGuire, 2015). Consequently, there is an urgent need to explore innovative diagnostic and therapeutic strategies that can improve patient outcomes. Currently, the application of cancer biomarkers has garnered significant attention from researchers, as these biomarkers hold the potential to facilitate early detection, improve treatment efficacy, and personalize therapeutic approaches, prompting further investigation into novel cancer biomarkers (Daassi et al., 2020).

Post-translational modification (PTM) is a complex mechanism that regulates cellular biological functions. It encompasses various types of modifications, including phosphorylation, acetylation, methylation, and ubiquitination, each of which has distinct biological roles. The ubiquitin proteasome system is the major pathway for intracellular protein degradation and relies on ATP supply to specifically degrade misfolded or damaged proteins (Zhang et al., 2025a). Among these, ubiquitination plays a crucial role in influencing protein-protein interactions, cellular localization, and substrate enzyme activity (Millar et al., 2019; Swatek and Komander, 2016; Zhang et al., 2025b). This modification is reversible; deubiquitinase catalyzes the removal of ubiquitin from substrate proteins, thus reversing the process (He et al., 2016; He et al., 2017). Ubiquitin-specific proteases (USPs) represent a significant subclass of deubiquitinases that are vital for maintaining protein homeostasis. They regulate various biological processes, such as the cell cycle, DNA repair, and signal transduction, by removing ubiquitin molecules from target proteins to prevent their degradation (Komander et al., 2009; Reyes-Turcu et al., 2009; Frappier and Verrijzer, 2011; Kee and Huang, 2016; Darling et al., 2017; Woo and Baek, 2019). Therefore, in-depth research on the specific role of deubiquitinase in tumors not only helps to reveal the molecular mechanisms of tumors but also may provide a basis for the discovery of new therapeutic targets, thereby promoting the development of tumor therapy.

Several studies have indicated that elevated expression of USP37 in various tumors is closely associated with patient prognosis, including colorectal cancer (Wu et al., 2025) renal cancer (Hong et al., 2020), breast cancer (Cao et al., 2023; Zha et al., 2016), and gastric cancer (Wu et al., 2021). As a key member of the USP deubiquitinase family, USP37 plays a critical role in the onset and progression of various tumors by regulating the stability of multiple oncogenic substrates, thereby influencing essential processes such as cell proliferation and survival. For example, USP37 enhances breast cancer progression by stabilizing estrogen receptor alpha (ERα) (Cao et al., 2023). In colorectal cancer, it promotes angiogenesis and metastasis by increasing the stability of beta-catenin (Wu et al., 2025). Additionally, USP37 regulates lung cancer cell proliferation and the Warburg effect by deubiquitinating and stabilizing c-Myc expression (Pan et al., 2015). Despite preliminary investigations into the role of USP37 in some tumors, there is currently a lack of pan-cancer analyses focusing on the USP37 gene. Accordingly, there is a need for additional investigations to uncover the USP37 regulatory functions and molecular mechanisms in a comprehensive cancer dataset. This discovery will offer new perspectives and insights into the molecular mechanisms underlying tumorigenesis and development, as well as aid in the formulation of therapeutic strategies.

In this study, we mainly conducted a comprehensive pan-cancer analysis of USP37 in expression levels, mutation status, patient prognosis, and immune infiltration using public databases. Furthermore, we performed functional experiments with pancreatic cancer tissues and cells to elucidate the functions of USP37. Based on these, our study suggests USP37 as a novel biomarker to predict prognosis and immune therapy response in diverse cancer types. Furthermore, this offers crucial insights for a deeper understanding of the role of USP37 in oncology.

## 2 Methods and materials

### 2.1 Data source

The UCSC Xena database (https://xenabrowser.net/datapages/) was utilized to obtain mRNA expression data and clinical information from the TCGA pan-cancer cohort and GTEx datasets. Transcriptomic profiles for 21 cancer cell lines were retrieved from the Cancer Cell Line Encyclopedia (CCLE) (https://sites.broadinstitute.org/ccle/). Genomic alterations of USP37 across 33 types of cancer were analyzed using the cBioPortal for Cancer Genomics tool (http://cbioportal.org). The subcellular localization of the USP37 protein was confirmed through data from the Human Protein Atlas (HPA) (https://www.proteinatlas.org/). The abbreviations for various cancer types are provided in Table 1.

**TABLE 1:** Abbreviations used in this pan-cancer analysis.

Abbreviations	Full Name
ACC	Adrenocortical carcinoma
BLCA	Bladder Urothelial Carcinoma
BRCA	Breast invasive carcinoma
CESC	Cervical squamous cell carcinoma and endocervical adenocarcinoma
CHOL	Cholangiocarcinoma
COAD	Colon adenocarcinoma
DLBC	Lymphoid Neoplasm Diffuse Large B-cell Lymphoma
ESCA	Esophageal carcinoma
GBM	Glioblastoma multiforme
HNSC	Head and Neck squamous cell carcinoma
KICH	Kidney Chromophobe
KIR	Kidney renal clear cell carcinoma
KIRP	Kidney renal papillary cell carcinoma
LAML	Acute Myeloid Leukemia
LGG	Lower Grade Glioma
LIHC	Liver hepatocellular carcinoma
LUAD	Lung adenocarcinoma
LUSC	Lung squamous cell carcinoma
MESO	Mesothelioma
OV	Ovarian serous cystadenocarcinoma
PAAD	Pancreatic adenocarcinoma
PCPG	Pheochromocytoma and Paraganglioma
PRAD	Prostate adenocarcinoma
READ	Rectum adenocarcinoma
SARC	Sarcoma
SKCM	Skin Cutaneous Melanoma
STAD	Stomach adenocarcinoma
TGC	Testicular Germ Cell Tumors
THCA	Thyroid carcinoma
THYM	Thymoma
UCEC	Uterine Corpus Endometrial Carcinoma
UCS	Uterine Carcinosarcoma
UVM	Uveal Melanoma

### 2.2 Gene set enrichment analysis

The hallmark gene set file (h.all.v7.4.symbols.gmt), consisting of 50 hallmark gene sets, was acquired from the Molecular Signatures Database (MSigDB, https://www.gsea-msigdb.org/gsea/index.jsp). This file was employed to determine the normalized enrichment score (NES) and false discovery rate (FDR) for differentially expressed genes (DEGs) between groups with high and low USP37 expression across different cancer types. Specifically, significance was assessed using permutation-based FDR. The “high USP37 expression” and “low USP37 expression” subgroups based on the median expression value of USP37 across all samples in the analyzed cohort in GSEA. Gene Set Enrichment Analysis (GSEA) was carried out using the R package “clusterProfiler”, and the findings were illustrated with a bubble plot created using the R package “ggplot2” (Yu et al., 2012).

### 2.3 Immunotherapy prediction analysis

Using the Sangerbox tool platform (http://past20.sangerbox.com/), we conducted a comprehensive Spearman correlation analysis to assess the statistical relationships between USP37 and established immunotherapy biomarkers across various cancer types. Key metrics, such as tumor mutation burden (TMB) and microsatellite instability (MSI), were included in the analysis, alongside the evaluation of correlations with other immune checkpoint-related genes. This investigation provides insights into USP37’s potential role in the tumor immune microenvironment and aids in assessing its viability as a target for immunotherapy (Shen et al., 2022).

### 2.4 Protein-protein interaction and prognostic analysis

The interaction network data involving USP37 comes from PPI research (https://genemania.org/search/). For prognostic analysis, we used data from UCSC (https://xenabrowser.net/). The standardized pan-cancer dataset, sourced from the TCGA prognostic study, includes overall survival (OS) and disease-specific survival (DSS) data. Using the survival and Survminer R software packages, the Cox regression model and Kaplan-Meier analysis were performed to examine the relationship between USP37 expression and prognosis. The log-rank test was used to compare the high-expression and low-expression subgroups, and a p-value less than 0.05 was considered statistically significant.

### 2.5 DNA repair genes and DNA methyltransferases analysis

We investigated the correlation between the expression of five mismatch repair (MMR) genes—MLH1, MSH2, MSH6, PMS2, and EPCAM—and USP37 using expression profile data from TCGA. These MMR genes are essential for preserving genomic stability and repairing DNA errors. Additionally, DNA methylation, a key epigenetic modification, has a significant impact on gene expression and is intricately linked to tumor development and progression (Martisova et al., 2021). To further understand the role of USP37 in relation to MMR gene expression, we explored their potential mechanisms in tumorigenesis and examined the influence of DNA methylation. Specifically, we analyzed the co-expression of USP37 with five DNA methyltransferases (DNMT1, TRDMT1, DNMT3A, DNMT3B, and DNMT3L) to assess the connection between USP37 and DNA methylation. The results of these analyses were visualized using heatmaps generated by the Sangerbox online platform (http://www.sangerbox.com/tool) (Shen et al., 2022).

### 2.6 Cell lines and cultures

Normal pancreatic ductal epithelial cells (H6C7, utilized as controls) and pancreatic cancer cell lines (AsPC-1, BxPC-3, SW 1990, PANC-1) were sourced from the American Type Culture Collection (Manassas, VA, USA). For cell culture, we employed Dulbecco’s Modified Eagle Medium (DMEM) provided by Gibco, Thermo Fisher (China), supplemented with 10% fetal bovine serum and 100 U/mL penicillin-streptomycin from Invitrogen (USA). The cells were grown under standard conditions at 37 °C in a humidified incubator with 5% CO_2_ to ensure optimal growth and maintenance.

### 2.7 Quantitative real-time (qRT)-PCR

Total RNA was extracted from cells or tissues using Trizol reagent (Invitrogen, USA). The RNA was then reverse-transcribed into complementary DNA (cDNA) using a reverse transcription kit. Subsequently, cDNA was amplified through quantitative real-time PCR (qRT-PCR) using SYBR Green qPCR Master Mix (Clontech Laboratories, USA), which contains DNA polymerase, primers, and SYBR Green dye. SYBR Green dye binds specifically to double-stranded DNA, emitting fluorescence proportional to the amount of DNA synthesized during amplification. The amplification was conducted on an Applied Biosystems® 7900HT Fast Real-Time PCR System (Thermo Fisher Scientific, USA). The PCR process involved denaturation, annealing, and extension phases. Fluorescence intensity was monitored in real-time during each cycle, enabling the quantification of the target gene relative to a housekeeping gene. Data were analyzed using the comparative Ct (ΔΔCt) method, which compares the threshold cycle (Ct) values of the target gene to those of the reference gene to determine relative expression levels. The following primers were used: for USP37, the forward primer sequence was 5′-TCTCTATTGACCTTCCTCGTAGG-3′ and the reverse primer sequence was 5′-TGCCTGACAAGAGCACACTTCC-3′; for GAPDH, the forward primer sequence was 5′-GTCTCCTCTGACTTCAACAGCG-3′ and the reverse primer sequence was 5′-ACCACCCTGTTGCTGTAGCCAA-3′.

### 2.8 Western blotting

Western blotting was utilized to evaluate the expression levels of target proteins in cell lysates. Cells were first harvested and lysed using RIPA buffer (Thermo Fisher Scientific, USA) supplemented with protease and phosphatase inhibitors (Roche, Switzerland). Protein concentrations in the lysates were determined with the BCA Protein Assay Kit (Pierce, USA). Equal quantities of protein (30 µg) were resolved by SDS-PAGE on 10% polyacrylamide gels and then transferred to PVDF membranes (Millipore, USA) using a semi-dry transfer apparatus (Bio-Rad, USA). The membranes were blocked with 5% non-fat dry milk in TBST (Tris-buffered saline with Tween 20) for 1 h at room temperature to reduce non-specific binding. Membranes were then incubated overnight at 4 °C with primary antibodies specific to the target proteins, diluted in 5% BSA in TBST. After thorough washing, the membranes were exposed to HRP-conjugated secondary antibodies (Thermo Fisher Scientific, USA) for 1 h at room temperature. Protein bands were visualized using ECL Western blotting Substrate (Bio-Rad, USA) and detected with a chemiluminescence imaging system (GE Healthcare, USA). Band intensities were quantified using ImageJ software (NIH, USA). The antibodies used were: USP37 (1:500, Proteintech, 18465-1-AP), α-Tubulin (1:1000, Proteintech, 66031-1-Ig).

### 2.9 Construction of lentivirus and cell infection

The USP37 human shRNA lentivirus and the USP37 overexpression lentivirus were both acquired from Genechem Company (Shanghai, China). The sequences for the USP37 human shRNA lentivirus are as follows: shUSP37#1: 5′-GCAGAAGATGATATTCCAGAA-3′; shUSP37#2: 5′-CGCTTTGCAAACCTGCTTATT-3′; and shNC: 5′-UUCUCCGAACGUGUCACGUTT-3′. For cell infection, target cells were initially seeded in 6-well plates at a density of 2 × 10^5^ cells per well. The following day, the cells were subjected to a 6-h starvation period by replacing the medium with serum-free DMEM to enhance viral uptake. After the starvation period, the lentiviral supernatant containing the virus was added to the cells. The cells were then incubated with the viral supernatant in a humidified incubator at 37 °C with 5% CO2 for 8 h. Subsequently, the viral-containing medium was removed and replaced with a fresh complete medium supplemented with 10% fetal bovine serum to facilitate cell recovery and proliferation. After a total incubation period of 48 h, the cells were selected using puromycin (2 μg/ml, Sigma-Aldrich, USA) to isolate those successfully transduced. Selection was maintained for 1–2 weeks to establish stable cell lines. The efficiency of transduction was assessed through fluorescence microscopy or PCR analysis, depending on the reporter gene used.

### 2.10 Cloning formation experiment

After processing, the cells were inoculated into a 6-well plate at a density of 2 × 10^3^ cells per well and cultured in a complete serum-containing medium for 2 weeks. Following this incubation period, the cells were washed three times with PBS to remove any residual culture medium. Subsequently, the cells were fixed with 4% paraformaldehyde (P1110, Solarbio) for 15 min to preserve cell morphology. The cells were then stained with crystal violet (C8470, Solarbio) for 2 h. Once staining was complete, the cells were washed again with PBS, allowed to air dry, and subsequently photographed and counted under a microscope.

### 2.11 CCK-8 assay

The processed cells were seeded into a 96-well plate at a density of 1 × 10^4^ cells per well. Following inoculation, the cells were incubated under appropriate conditions for 24 h to ensure optimal adhesion and growth. The experiment was then conducted according to the manufacturer’s instructions for the CCK-8 reagent kit. Specifically, CCK-8 reagent was added to each well, and after a defined incubation period, the absorbance was measured at 450 nm using a microplate reader. This procedure assesses cell survival and proliferation, with changes in absorbance indicating variations in cell growth.

### 2.12 EdU assays

To conduct EdU (5-ethynyl-2′-deoxyuridine) detection, treated cells were initially seeded into 6-well plates and cultured to achieve 70%–80% confluence, ensuring stable cell growth during the treatment. EdU solution was then added to the cell culture medium at a concentration of 10 µM and incubated for 6 h at 37 °C in a 5% CO2 environment. This incubation allows EdU to be effectively incorporated into newly synthesized DNA. Following incubation, cells were washed three times with PBS to remove any unbound EdU. The cells were then fixed with 4% paraformaldehyde for 15 min to preserve cellular morphology and subsequently washed three times with PBS. To enhance EdU detection sensitivity, the cells were permeabilized with 0.5% Triton X-100 for 5 min, which removes lipids from the cell membrane and facilitates better penetration of staining reagents. After permeabilization, EdU staining was performed according to the manufacturer’s instructions, with incubation at room temperature for 60 min. Post-staining, the cell nuclei were counterstained with DAPI for 5 min to allow for visual differentiation under a microscope. Cells were then washed with PBS to remove excess staining solution. Finally, images were captured using a microscope, and the percentage of EdU-positive cells was calculated through image analysis to assess cell proliferation.

### 2.13 Tumorigenicity assays and bioluminescence imaging

Tumorigenicity assays and bioluminescence imaging were performed using pancreatic cancer cells that were stably transduced with the firefly luciferase gene, allowing for consistent monitoring of tumor growth through bioluminescent imaging. For the *in vivo* tumorigenicity experiments, 2 × 10^6^ cells suspended in 100 µL of PBS were subcutaneously injected into the flanks of male BALB/c-nu/nu nude mice. To detect bioluminescent signals *in vivo*, the mice were anesthetized with isoflurane and imaged using the IVIS Lumina Series III system (PerkinElmer, MA, USA). All animal procedures were approved by the Ethics Committee for Animal Experiments at First Hospital of Putian City.

### 2.14 Ethical support

Pancreatic cancer tissue and adjacent non-tumor tissue samples were collected from patients at the First Hospital of Putian City. In accordance with the Declaration of Helsinki, all participants provided written informed consent. This study’s use of animals was approved by the Ethics Committee of First Hospital of Putian City. (Ethics No. 2024–192). All methods were performed in accordance with the relevant guidelines and regulations.

### 2.15 Immunohistochemical (IHC)

Tissue sections were initially placed in a 60 °C oven for an hour to dry, followed by cooling to ambient temperature. The paraffin was then removed using a dewaxing solution, and the sections were rehydrated through a series of ethanol gradient solutions. After washing with PBS buffer, endogenous peroxidase activity was blocked by treating the sections with 3% hydrogen peroxide for 10 min. To prevent non-specific binding, the sections were incubated with 5% normal goat serum. Next, the sections were exposed overnight at 4 °C to an anti-USP37 antibody (1:250). Following the antibody incubation, the sections were washed with PBS to eliminate any unbound antibodies. A secondary antibody solution was then applied, and the sections were incubated at room temperature for 30 min. After another PBS wash, the sections were stained with DAB (3,3′-diaminobenzidine) until the desired intensity was achieved. The staining was followed by counterstaining with hematoxylin and dehydration through a gradient of ethanol solutions. Finally, the sections were mounted using a mixture of neutral resin and xylene. Stained sections were examined and photographed under a microscope for further analysis.

### 2.16 Statistical analysis

All results are expressed as mean ± standard deviation (SD) and were analyzed using GraphPad Prism 8.0, based on at least three independent experiments. Differences between groups were assessed using two-tailed Student's t-tests and analysis of variance (ANOVA). Statistical significance was determined at a p-value of less than 0.05.

## 3 Results

### 3.1 USP37 is aberrantly expressed in PAAD tissues

**FIGURE 1:**
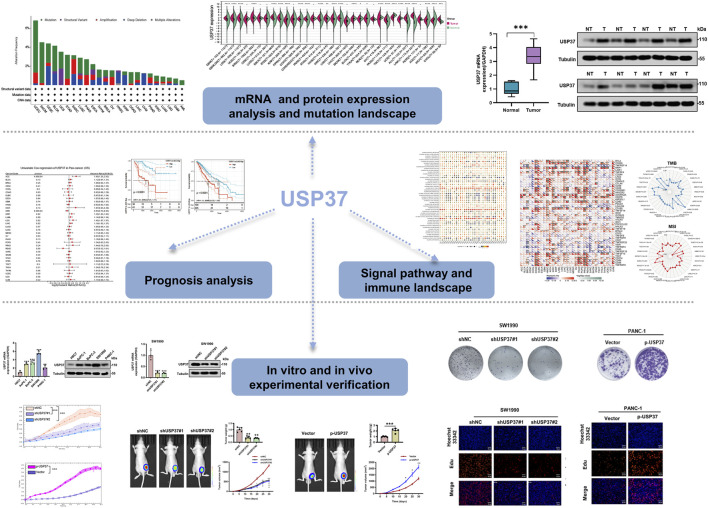
Setup of this study.

This study conducted a systematic analysis of USP37 across various cancer types using several public databases, including TCGA, to investigate its potential role in tumor development and prognosis. The investigation covered gene expression differences among different cancers, examined the relationship between USP37 expression and patient survival, explored its mutation profile, and assessed its link to immune infiltration and key cellular signaling pathways. By integrating these analyses, the study seeks to clarify USP37’s biological functions in cancer progression and its potential as a therapeutic target and prognostic marker. The research workflow is depicted in Figure 1.

**FIGURE 2:**
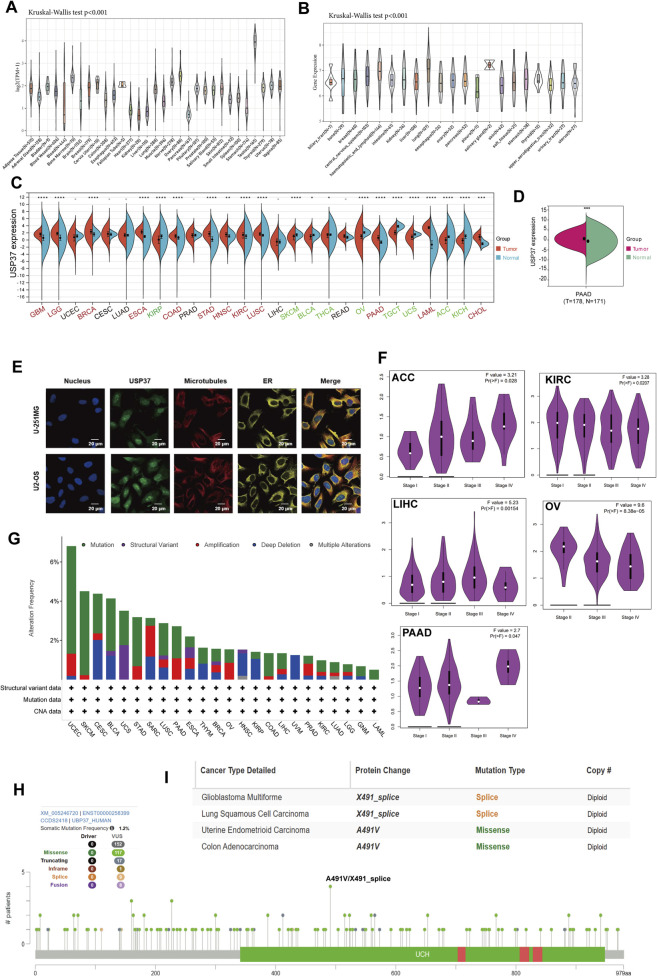
Overview of USP37 details. **(A)** The expression levels of USP37 in normal tissues. **(B)** The USP37 expression in tumor cell lines. **(C)** The expression levels of USP37 in tumor versus normal tissues for each cancer type were analyzed based on integrated data from TCGA and GTEx datasets. **(D)** The USP37 levels between PAAD and normal pancreatic tissue. **(E)** Immunofluorescence images showing USP37 protein, nucleus, ER, microtubules, and their merged views in U-251MG, and U2-OS cell lines. **(F)** Association between USP37 expression and tumor stage in ACC, KIRC, LIHC, OV, and PAAD. **(G)** Analysis of USP37 alteration frequencies in pan-cancer studies based on data from the cBioPortal database. **(H,I)**. The mutation sites of USP37 and the highest alteration frequency (A491V/X491_splice) in TCGA tumors are displayed.

We began by examining USP37 gene expression across various tissues using the GTEx dataset, which revealed considerable heterogeneity. The highest expression of USP37 was observed in testicular tissue (Figure 2A). Similarly, CCLE data analysis demonstrated significant variability in USP37 expression among different cell lines (p < 0.001), consistent with the GTEx findings (Figure 2B). To further explore USP37 expression in cancers, we analyzed mRNA levels from TCGA and GTEx datasets across multiple tumor types. High USP37 expression was detected in GBM, LGG, BRCA, ESCA, COAD, STAD, HNSC, KIRC, LUSC, PAAD, LAML, and CHOL, while lower expression was observed in KIRP, SKCM, BLCA, THCA, OV, TGCT, UCS, ACC, and KICH (Figure 2C). Notably, both USP37 mRNA levels were significantly higher in PAAD tissues compared to normal controls (Figure 2D). Immunofluorescence (IF) imaging revealed that the USP37 protein predominantly localized in the nuclei of U2-OS and U-251MG tumor cells, with minimal cytoplasmic expression (Figure 2E). Finally, using the “Pathological Stage Plot” function in HEPIA2, we analyzed the correlation between USP37 expression and pathological stages in ACC, KIRC, LIHC, OV, and PAAD (Figure 2F). In summary, USP37 shows varying expression across tissues and cancers, with higher levels in several tumors, especially PAAD, and is mainly localized in the nucleus of tumor cells.

Analysis of USP37 genomic alterations revealed its mutations in several tumor types. The highest mutation frequency was observed in UCEC, affecting over 6% of patients, with “mutation” being the most frequent alteration, followed by “amplification” (Figure 2G). Figure 2H provides a detailed overview of the types, locations, and frequencies of USP37 genetic alterations. Of the 152 identified genetic variations, “Missense” mutations were the most prevalent, particularly the A491V/X491_splice mutation in the UCH domain. The X491_splice mutations, found in cancers such as GBM and LUSC led to a splice mutation at position 491 of the USP37 protein. Additionally, A491V mutations were identified in UCEC and COAD, resulting in a missense mutation at the same position within the *USP37* gene (Figure 2I).

### 3.2 Association analysis between USP37 expression and prognosis

**FIGURE 3:**
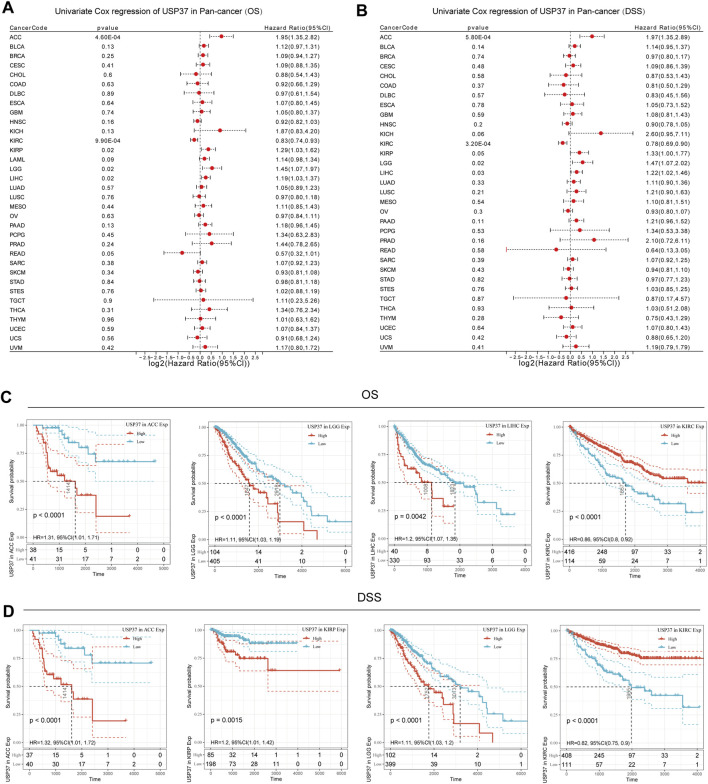
Prognostic analysis of USP37 in pan-Cancer. **(A)** The forest plot displays the association between USP37 expression and overall survival (OS) using the univariate Cox regression method. **(B)** The forest plot displays the association between USP37 expression and DSS using the univariate Cox regression method. **(C)** Kaplan‒Meier OS curves of USP37 in ACC, LGG, LIHC, and KIRC. **(D)** Kaplan‒Meier DSS curves of ACC, KIRP, LGG, and KIRC.

We utilized the Kaplan-Meier method along with univariate Cox regression analysis to assess the prognostic significance of USP37 in a pan-cancer context. The forest plot (Figure 3A) indicated that lower USP37 expression was linked to improved overall survival (OS) in certain cancers, including ACC (HR = 1.95, 95% CI [1.35–2.82], p = 0.00046), KIRP (HR = 1.29, 95% CI [1.03–1.62], p = 0.02), LGG (HR = 1.45, 95% CI [1.07–1.97], p = 0.02) and LIHC (HR = 1.19, 95% CI [1.03–1.37], p = 0.02). In contrast, higher USP37 expression was associated with better OS in KIRC (HR = 0.83, 95% CI [0.74–0.93], p = 0.00099). The analysis also extended to disease-specific survival (DSS), where reduced USP37 expression corresponded to better DSS outcomes in ACC (HR = 1.97, 95% CI [1.35–2.89], p = 0.00058), LGG (HR = 1.47, 95% CI [1.07–2.02], p = 0.02), and LIHC (HR = 1.22, 95% CI [1.02–1.46], p = 0.03). Conversely, USP37 overexpression was related to worse DSS in KIRC (HR = 0.78, 95% CI [0.69–0.90], p = 0.00032) (Figure 3B). Kaplan-Meier survival curves for ACC, LGG, LIHC, KIRC showed that higher USP37 expression correlated with poorer OS in ACC, LGG, and LIHC, while being associated with better OS in KIRC (Figure 3C). Similarly, elevated USP37 expression was linked to shorter DSS in ACC, KIRP, and LGG, but prolonged DSS in KIRC patients (Figure 3D). Univariate and multivariate Cox regression analyses of overall survival are presented in Table 2. Overall, these results suggest that USP37 may serve as a prognostic predictor across various cancers.

**TABLE 2:** Univariate and multivariate Cox regression analysis of OS.

Characteristics	Total(N)	Univariate analysis		Multivariate analysis	
		HR (95% CI)	P Value	HR (95% CI)	P Value
Pathologic T stage	370				
T1	183				
T2	94	1.431 (0.902–2.268)	0.128	0.000 (0.000 - Inf)	0.994
T3	80	2.674 (1.761–4.060)	<0.001	0.766 (0.103–5.674)	0.794
T4	13	5.386 (2.690–10.784)	<0.001	1.332 (0.151–11.786)	0.797
Pathologic N stage	258				
N0	254				
N1	4	2.029 (0.497–8.281)	0.324		
Pathologic M stage	272				
M0	268				
M1	4	4.077 (1.281–12.973)	0.017	5606423.6583 (0.000 - Inf)	0.999
Pathologic stage	349				
Stage I	173				
Stage II	86	1.417 (0.868–2.312)	0.164	7506516.6440 (0.000 - Inf)	0.994
Stage III	85	2.734 (1.792–4.172)	<0.001	4.079 (0.543–30.667)	0.172
Stage IV	5	5.597 (1.726–18.148)	0.004	0.000 (0.000 - Inf)	0.999
USP37	373				
Low	186				
High	187	1.387 (0.980–1.962)	0.065	1.145 (0.736–1.781)	0.547

### 3.3 Gene set enrichment analysis (GSEA) and DNA methylation of USP37 in pan-cancer

**FIGURE 4:**
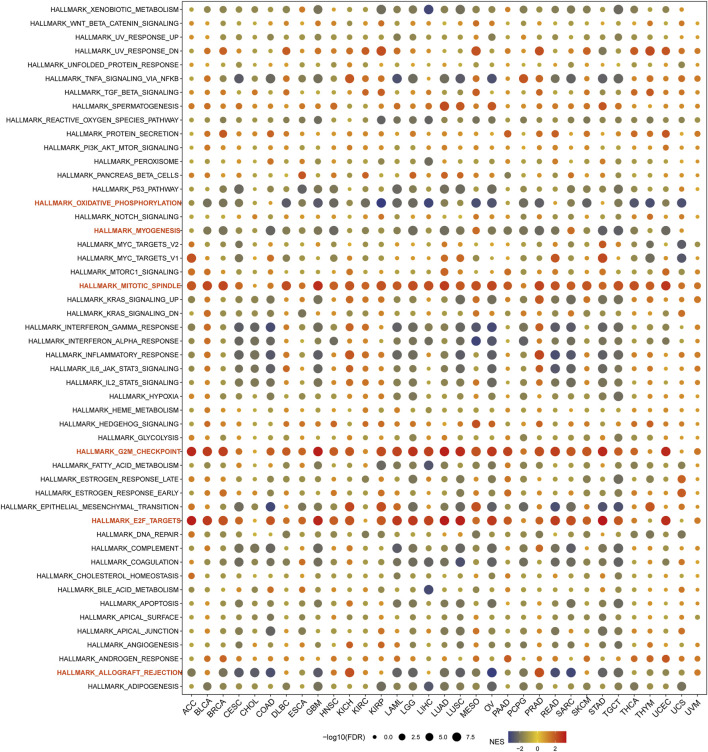
Gene Set Enrichment Analysis (GSEA) of USP37 in Pan-Cancer. The circle size represents the FDR value of the enriched term in each cancer, and the color indicates each term’s normalized enrichment score (NES).

To identify USP37-associated cancer hallmarks, we performed gene set enrichment analysis (GSEA) using differentially expressed genes (DEGs) between low and high USP37 expression subgroups for each cancer type. Our analysis revealed that USP37 expression was significantly associated with oxidative phosphorylation, myogenesis, mitotic spindle, G2M checkpoint, E2F targets, and allograft rejection pathways. In the pathway enrichment analysis, USP37 expression was found to be positively correlated with key cell cycle-related pathways, including the mitotic spindle, G2M checkpoint, and E2F targets in most majority of cancers (Figure 4).

**FIGURE 5:**
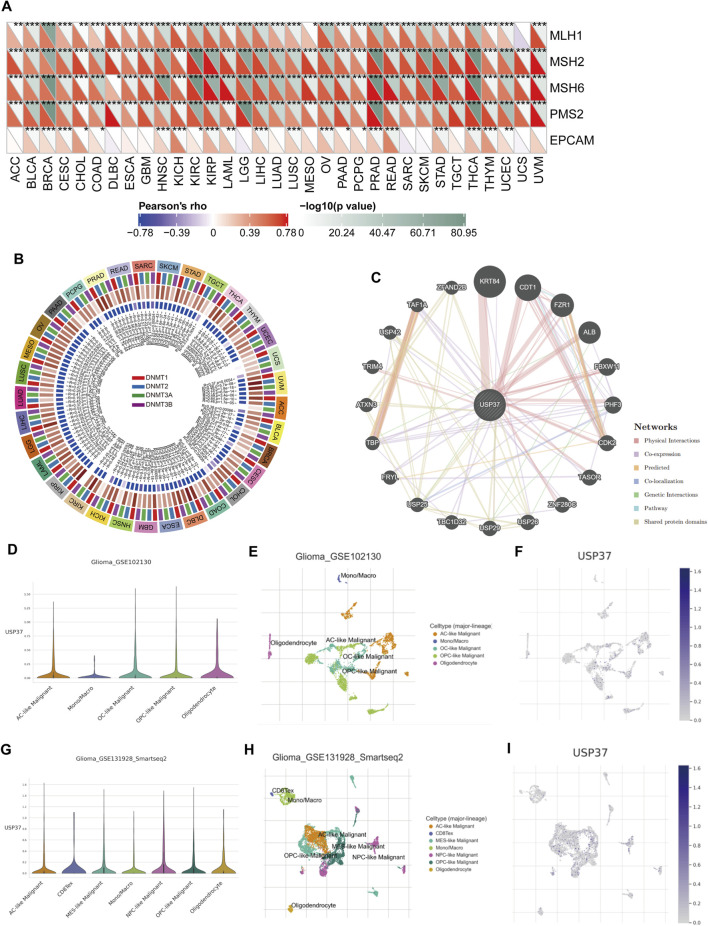
The association of USP37 expression with expression levels of five MMR genes and four DNA methyltransferases. **(A)** Spearman correlation analysis of USP37 expression with the expression levels of five MMR genes across cancer types. **(B)** Spearman correlation analysis of USP37 expression with four DNA methyltransferases across various cancers, red: DNMT1, blue: DNMT2, green: DNMT3A, purple: DNMT3B. **(C)** The gene-gene and protein-protein interaction network was generated by using GeneMANIA. **(D-H)** Main cell types expressing USP37 in the Glioma-GSE102130 cohort **(D–F)** and Glioma-GSE131928 cohort **(G,H)**.

MMR (mismatch repair) is an essential cellular process that ensures the correction of DNA replication errors, maintaining genetic stability in all organisms. When this repair mechanism fails, it can result in mutations and genetic abnormalities, contributing to the development of various diseases, particularly cancer (Pecina-Slaus et al., 2020; Olave and Graham, 2022; Emiloju et al., 2023). A study was conducted to investigate the relationship between USP37 expression levels and mutations in five key MMR genes to explore the role of USP37 in tumorigenesis. The findings demonstrated a significant positive association between USP37 and MLH1, MSH2, MSH6, PMS2, and EPCAM across most cancer types. However, it is important to note that, compared to the other four MMR genes, USP37 exhibits a weaker positive correlation with EPCAM and shows no correlation in certain tumors, including ACC, DLBC, ESCA, GBM, LGG, LUAD, MESO, SARC, SKCM, TGCT, UCS, and UVM (Figure 5A). Abnormal DNA methylation is one of the key factors in tumor development, which promotes tumor progression by regulating gene expression. It can lead to silencing of tumor suppressor genes or activation of oncogenes, thereby promoting cell proliferation, inhibiting apoptosis, and enhancing invasiveness. Therefore, DNA methylation, as a common epigenetic variation, has become an important biomarker for cancer diagnosis, treatment, and prognosis evaluation (Papanicolau-Sengos and Aldape, 2022; Zhu et al., 2023). The results indicated that USP37 was significantly associated with at least one of the four methyltransferase genes (DNMT1, DNMT2, DNMT3A, and DNMT3B) in various cancer types, except UCS (Figure 5B).

Next, we utilized the GeneMANIA online program to create a PPI network for USP37, which is displayed in Figure 5C, to investigate the probable processes by which USP37 played a role in cancer carcinogenesis. USP37 demonstrated significant physical interactions with KRT84, CDT1, FZR1, and ALB, as illustrated in Figure 5C. Research has shown that reducing CDT1 expression can significantly inhibit the malignant behavior of HCC cells (Yao et al., 2023), which indicates that USP37 may influence tumor progression by modulating the activity of CDT1 and other interacting proteins, requiring further investigation.

### 3.4 Single-cell analysis of USP37 expression across multiple cell types

To explore the mechanisms by which USP37 influences the tumor immune microenvironment, public single-cell RNA-seq datasets were analyzed to assess USP37 expression across various immune cell types. The heatmap revealed that USP37 is expressed in immune cells, particularly T lymphocytes—including CD4 Tconv, Treg, CD8 T, and CD8 Tex cells—followed by innate immune cells such as NK cells, dendritic cells (DCs), and monocytes/macrophages. The expression and distribution of USP37 across different immune cells in various tumors are shown in Supplementary Figure S1. Specifically focusing on gliomas and considering the level of USP37 expression, we demonstrated its distribution in immune cells. Our analysis indicated that in gliomas_GSE102130, USP37 is primarily expressed in Ac-like malignant cells, OC-like malignant cells, OPC-like malignant cells, oligodendrocytes, and monocytes/macrophages (Figures 5D–F). In gliomas_GSE1311928_Smartseq2, USP37 is expressed in Ac-like malignant cells, CD8Tex, MES-like malignant cells, monocytes/macrophages, NPC-like malignant cells, OPC-like malignant cells, and oligodendrocyte (Figures 5G–I). This implies that USP37 might play a role in regulating the interactions between immune cells and tumor cells, potentially affecting glioma progression and immune evasion. Nevertheless, additional research is needed to clarify the specific functional roles of USP37 in these cellular processes.

### 3.5 Connections between USP37, immune regulators, TMB, and MSI

**FIGURE 6:**
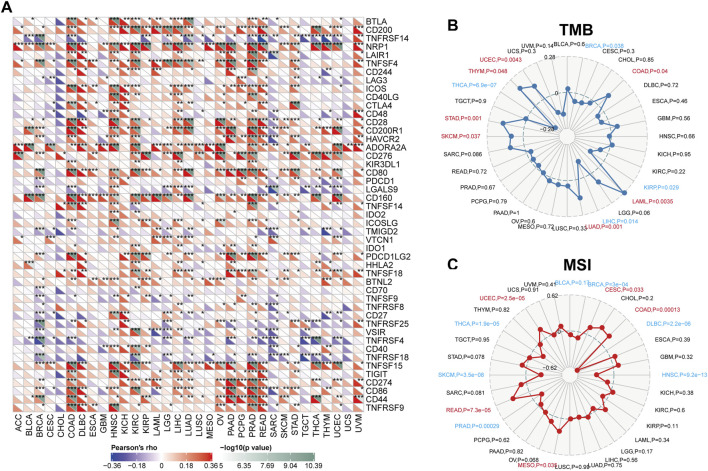
Correlations between USP37 expression and immunity, encompassing immune marker sets, tumor mutation burden (TMB), and microsatellite instability (MSI) across various cancers. **(A)** The Spearman correlation heatmap illustrates the relationships between USP37 expression and various immune regulators across pan-cancer types. Positive correlations are depicted in red, while negative correlations are shown in blue. **(B)** The correlations between USP37 expression and TMB across pan-cancer types. **(C)** The correlations between USP37 expression and MSI across pan-cancer studies.

Figure 6A illustrates the associations between USP37 and 47 immune regulators across various cancer types. Our analysis revealed that USP37 exhibits a strong positive correlation with most immune regulators in cancers such as HNSC, KIRC, LIHC, PRAD, and READ while showing a strong negative correlation with most immune regulators in SARC. Additionally, USP37 demonstrated a significant positive relationship with ADORA2A, CD160, TNFSF15 and NRP1 across the majority of cancers. To assess USP37’s potential as a predictor of immune checkpoint inhibitor (ICI) efficacy, we evaluated the correlation between USP37 expression and both TMB and microsatellite instability (MSI).

**FIGURE 7:**
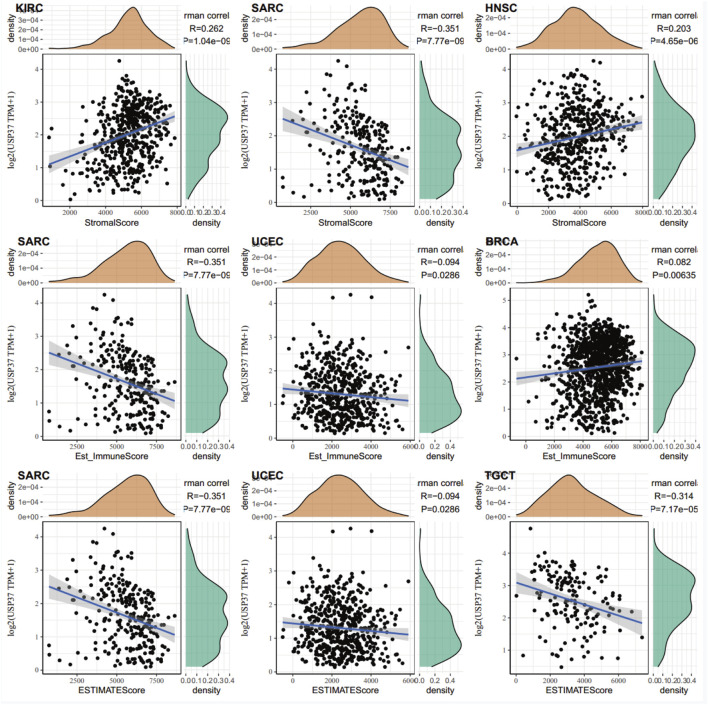
Top three cancers by ImmuneScore, StromalScore, and ESTIMATEScore, respectively.

Positive correlations between tumor mutation burden TMB and USP37 expression were observed in UCEC, THYM, STAD, SKCM, LUAD, LAML, and COAD. In contrast, negative correlations were identified in THCA, LIHC, KIRP, and BRCA (Figure 6B). For microsatellite instability (MSI), positive associations with USP37 expression were found in UCEC, READ, MESO, COAD, and CESC, while negative correlations were observed in DLBC, THCA, SKCM, PRAD, HNSC, DLBC, and BRCA (Figure 6C). These results suggest that USP37 could be a potential predictor of ICI efficacy in these cancers. Furthermore, the top three tumors showing the strongest correlations with USP37 expression were KIRC, SARC, and HNSC based on the StromalScore; SARC, UCEC, and BRCA based on the Estimated Immune Score; and SARC, UCEC, and TGCT based on the ESTIMATE Score (Figure 7). Overall, these findings highlight a robust association between USP37 expression and immune infiltration levels in these cancers.

### 3.6 USP37 is highly expressed in the clinical PAAD tissues

**FIGURE 8:**
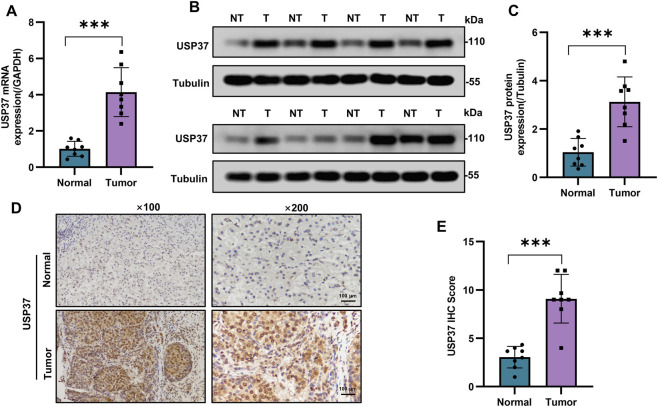
The USP37 expression levels in PAAD. **(A)** The USP37 mRNA expression in PAAD and normal pancreatic tissue. Data represent mean ± SD of independent experiments and were statistically analyzed with Student’s t-test, n = 8, ***P < 0.001. **(B)** and **(C)** The representative graph **(B)** and quantitative analysis **(C)** of the expression level of USP37 protein in PAAD and normal pancreatic tissue. Data represent mean ± SD of independent experiments and were statistically analyzed with Student’s t-test, n = 8, ***P < 0.001. **(D)** and **(E)** Immunohistochemical experiments were used to detect the protein expression of USP37 in PAAD and normal pancreatic tissue (magnification ×100, inset magnification ×200). Scale bar, 100 μm. Data represent mean ± SD of independent experiments and were statistically analyzed with Student’s t-test, n = 8, ***P < 0.001.

Next, we performed qRT-PCR and Western blot assays on clinical PAAD samples to validate the expression levels of USP37 in comparison to adjacent non-tumor tissues. The results showed that USP37 mRNA and protein expression levels are significantly increased in PAAD, consistent with the bioinformatics analysis. (Figures 8A–C). Additionally, IHC analyses demonstrated that USP37 was highly expressed in PAAD tissues (Figures 8D,E). These findings confirmed the elevated expression of USP37 in PAAD tissues, supporting its potential role in tumor progression and aligning with bioinformatics predictions.

### 3.7 Aberrant overexpression of USP37 promotes cell proliferation and tumorigenesis in pancreatic cancer

**FIGURE 9:**
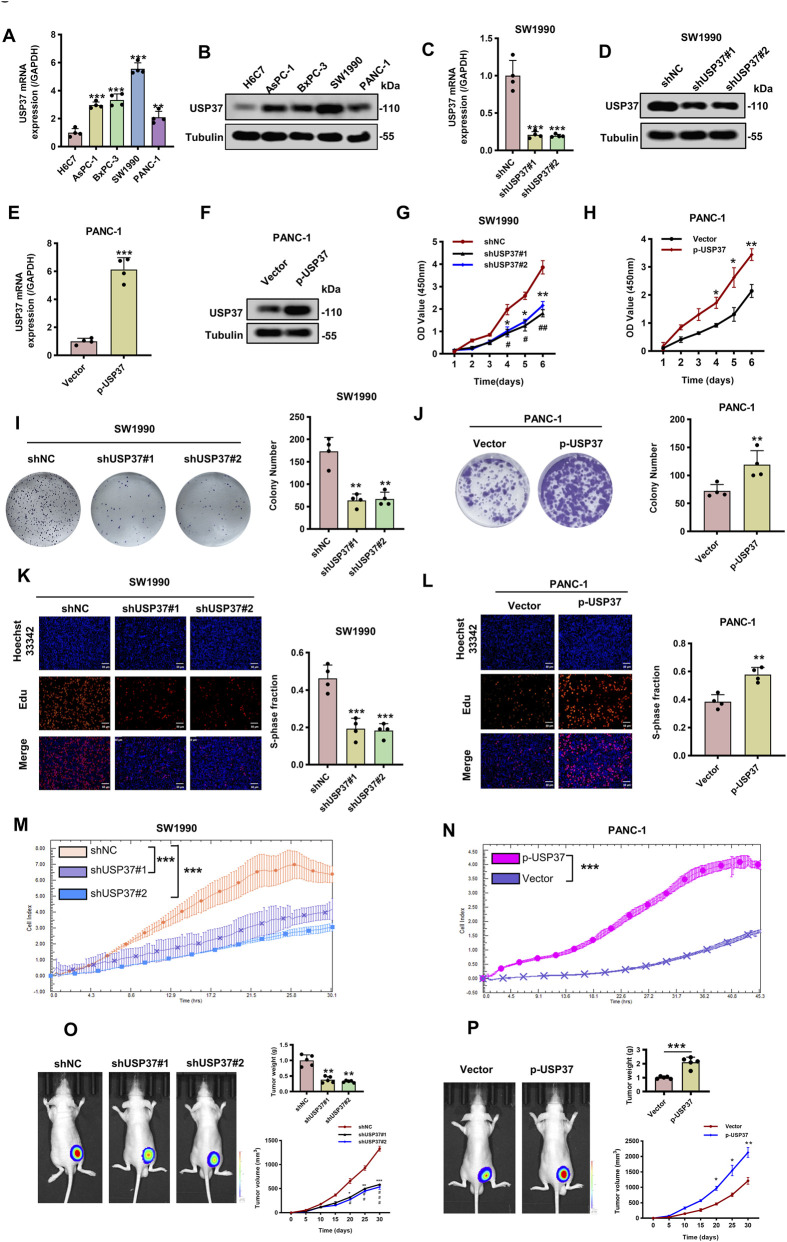
Knockdown of USP37 inhibits the proliferation of pancreatic cancer cells. **(A)** and **(B)** The mRNA **(A)** and protein **(B)** levels of USP37 in four pancreatic cancer cells and the immortalized H6C7 cell lines. Data represent mean ± SD of experiments and were statistically analyzed with one-way analysis of variance (ANOVA), n = 4, **P < 0.01, ***P < 0.001. **(C)** and **(D)** The mRNA **(C)** and protein **(D)** levels of USP37 were assessed in SW1990 transfected with shNC or shUSP37 by Western blotting assay. α-Tubulin was used as a loading control. Data represent mean ± SD of experiments and were statistically analyzed with one-way analysis of variance (ANOVA), n = 4, ***P < 0.001. **(E)** and **(F)** The mRNA **(E)** and protein **(F)** levels of USP37 were assessed in PANC-1 cells transfected with vector or p-USP37 plasmid by Western blotting assay. α-Tubulin was used as a loading control. Data represent mean ± SD of experiments and were statistically analyzed with Student’s t-test, n = 4, ***P < 0.001. **(G)** and **(H)** CCK-8 assay showing proliferation ability of pancreatic cancer cells following knockdown **(G)** or overexpressing **(H)** USP37, Data represent mean ± SD of experiments and were statistically analyzed with one-way analysis of variance (ANOVA) or Student’s t-test, n = 4, *P < 0.05, **P < 0.01. **(I)** and **(J)** Representative images (left) and quantification (right) of colony formation assays of pancreatic cancer cells transfected with shUSP37 **(I)** or p-USP37 **(J)**. Data represent mean ± SD of experiments and were statistically analyzed with one-way analysis of variance (ANOVA) or Student’s t-test, n = 4, **P < 0.01. **(K)** and **(L)** Representative images (left) and quantification (right) of EdU assays of pancreatic cancer cells transfected with shUSP37 **(K)** or p-USP37 **(L)**. Data represent mean ± SD of experiments and were statistically analyzed with one-way analysis of variance (ANOVA) or Student’s t-test, n = 4, **P < 0.01, ***P < 0.001. **(M)** and **(N)** RTCA assays were conducted to assess the proliferative ability of SW 1990 **(M)** and PANC-1 **(N)** cells transfected with USP37 knockdown and overexpression. **(O)** and **(P)** SW1990/shUSP37 cells **(O)** or PANC-1/p-USP37 cells **(P)** were subcutaneously injected into nude mice, and tumor volumes were measured on the indicated days; at the experimental endpoint, tumors were dissected, photographed, and weighed. n = 6, *P < 0.05, **P < 0.01, ***P < 0.001. ^#^ *P* < 0.05, ^##^ *P* < 0.01, ^###^ *P* < 0.001, “*” refers to the comparison between shNC and shUSP37#1 and “^#^” refers to the comparison between shNC and shUSP37#2.

The consistent overexpression of USP37 in PAAD and cell lines prompted us to investigate its potential oncogenic role in PAAD. To explore the role of USP37 in pancreatic cancer (PC) cell proliferation, we conducted a series of assays. First, we performed qRT-PCR and Western blot analyses to measure the mRNA and protein levels of USP37 in the human pancreatic duct epithelial cell line H6C7 and four PC cell lines: AsPC-1, BxPC-3, SW 1990, and PANC-1. The results showed that USP37 expression was highest in SW1990 cells and lowest in PANC-1 cells compared to H6C7 (Figures 9A,B, Supplementary Figure S2A). We then used USP37-targeting shRNA vectors to knock down USP37 expression in SW1990 cells, which exhibit high endogenous levels of USP37 (Figures 9C,D, Supplementary Figure S2B). Conversely, we generated USP37-overexpressing PANC-1 cells, which have relatively low endogenous USP37 levels (Figures 9E,F, Supplementary Figure S2C). Silencing USP37 in SW1990 cells significantly suppressed cell growth (Figure 9G), while USP37 overexpression in PANC-1 cells significantly enhanced cell proliferation, as demonstrated by CCK-8 assays (Figure 9H). Consistent with these findings, colony formation and EdU assays revealed that USP37 knockdown reduced cell proliferation in SW1990 cells (Figures 9I,L), whereas USP37 overexpression increased cell proliferation in PANC-1 cells (Figures 9J,K). Consistently, RTCA data indicated that USP37 knockdown significantly inhibited the proliferation of SW1990 cells, whereas its overexpression enhanced the proliferation of PANC-1 cells (Figures 9M,N). In conclusion, these findings suggest that USP37 can promote the proliferation of PC cells *in vitro*.

Finally, to evaluate the effect of USP37 on tumorigenesis *in vivo*, we performed subcutaneous xenograft assays in nude mice using USP37-knockdown SW1990 cells. After 4 weeks of growth, a significant reduction in tumor size and weight was observed (Figure 9O). In contrast, USP37-overexpressing PANC-1 cells showed markedly increased tumor growth and weight compared to controls (Figure 9P). Collectively, these data indicate that USP37 plays a crucial oncogenic role in promoting PC cell growth and tumorigenicity.

## 4 Discussion

The findings of this study provide significant insights into the role of USP37 in PAAD, highlighting its potential as a biomarker and therapeutic target. We firstly observed that USP37 is aberrantly expressed in PAAD tissues, suggesting that it could contribute to cancer progression. Aberrant overexpression in PAAD was validated in multiple ways, including both *in vitro* and *in vivo* models. This overexpression correlated with aggressive cell proliferation and increased tumorigenesis in murine models, underscoring USP37’s critical role in promoting malignancy in pancreatic cancer. These findings are consistent with previous reports in other tumors suggesting that USP37 (Cao et al., 2023; Wu et al., 2021; Chauhan et al., 2023; Stromberg et al., 2021; Hernandez-Perez et al., 2016), as a deubiquitinating enzyme, may stabilize proteins involved in cell cycle progression and survival, contributing to uncontrolled cell proliferation, and plays a multifaceted role in cancer progression. For instance, USP37 promotes breast cancer progression by stabilizing ERα through deubiquitination (Cao et al., 2023). In gastric cancer, USP37 enhances cell proliferation and migration by deubiquitinating and stabilizing Snail1 in a PLAGL2-dependent manner (Wu et al., 2021). Additionally, USP37 contributes to chemoresistance in hepatocellular carcinoma (HCC) by maintaining NRF2 protein stability (Zhang S. et al., 2025). Beyond oncogenic signaling, USP37 also safeguards genomic integrity by deubiquitinating replication protein A (RPA) at stalled replication forks, thereby preventing RPA hyperaccumulation, depletion, and subsequent DNA double-strand breaks (Tang et al., 2025). The evidence suggests that targeting USP37 could provide a promising strategy for curbing pancreatic tumor growth.

To further investigate the prognostic significance of USP37, we conducted an association analysis with patient survival data. Our results indicated that from both OS and DSS analyses were consistent, indicating that USP37 is a significant prognostic factor across a wide range of cancers. Specifically, high USP37 expression was linked to poor prognosis in ACC, KIRP, LGG, and LIHC, as shown in Kaplan-Meier survival curves. These curves demonstrated a clear association between elevated USP37 levels and worse patient outcomes in these cancer types. In contrast, increased USP37 expression in KIRC was associated with a more favorable prognosis. Previous studies have also highlighted USP37’s high expression in breast cancer stem cells, correlating with a poor prognosis in breast cancer patients (Qin et al., 2018). Research indicates that the immunosuppressive tumor microenvironment profoundly drives the occurrence, development, and treatment of pancreatic cancer, making its targeting central to immunotherapy (Lavin and Mc Gee, 2015; Yee, 2016). Significantly, while our findings and existing literature establish USP37 as a promoter of tumorigenesis and metastasis in cancers like LIHC (Zhang S. et al., 2025), its association with favorable prognosis in KIRC presents a compelling tissue-specific paradox. This suggests that the functional consequences of USP37 dysregulation maybe be critically dependent on the cellular and microenvironmental context of the specific tumor type. In KIRC, characterized by frequent VHL inactivation and constitutive HIF signaling, USP37 may preferentially target and stabilize substrates that exert tumor-suppressive or immunomodulatory functions (Hong et al., 2020). Notably, other USP family members—including USP22, USP10, and USP32—have been associated with adverse prognosis in various malignancies, including pancreatic ductal adenocarcinoma (Ning et al., 2014; Wang et al., 2024; Li et al., 2023), non-small cell lung cancer (Li et al., 2024). Taken together, these findings suggest that USP37 could be a valuable prognostic biomarker in cancer, offering insight into patient outcomes and aiding in the development of personalized therapeutic strategies. Moreover, exploring the molecular mechanisms by which USP37 influences patient survival could unveil therapeutic pathways for targeting this gene in aggressive PAAD subtypes.

The GSEA analysis reveals that USP37 is closely linked to several key pathways, including oxidative phosphorylation, myogenesis, mitotic spindle, G2M checkpoint, E2F targets, and allograft rejection. Notably, oxidative phosphorylation, myogenesis, and allograft rejection pathways show a negative correlation with USP37 expression across most tumor types, while its expression is positively associated with the mitotic spindle, G2M checkpoint, and E2F target pathways. These findings suggest that USP37 may exert distinct roles in different cancer types, potentially through varying mechanisms. Numerous previous studies have highlighted the critical roles of immune-related pathways in predicting prognosis, assessing immune infiltration, and evaluating responses to immunotherapy in PAAD patients (Li et al., 2022; Sun et al., 2023; Gu et al., 2021; Su et al., 2022; Chen et al., 2021). These pathways are not only essential for understanding the tumor microenvironment but also serve as important indicators for clinical outcomes and treatment efficacy. Our findings are consistent with these observations, reinforcing the significance of immune modulation in PAAD and further supporting the potential of immune-related pathways as biomarkers for prognosis and therapeutic strategies in this aggressive malignancy. Additionally, DNA methylation patterns suggest that the expression of USP37 may be regulated epigenetically, contributing to its aberrant expression in PAAD and potentially other cancers. Relevant literature has also reported that G9a, a histone methyltransferase (HMT), promotes methylation of histone H3 at lysine 9 (H3K9), including mono-, di-, and trimethylation, at the USP37 promoter, thereby repressing its gene expression in neural cancer (Dobson et al., 2017). These findings underscore the broader involvement of USP37 in oncogenic pathways across various cancers and highlight DNA methylation as a key regulatory mechanism influencing its activity.

Furthermore, our study’s exploration of USP37’s relationship with immune regulators, TMB and MSI emphasizes its possible role in shaping the immune landscape of PAAD. We found correlations between USP37 expression and key immune modulators, suggesting that it might contribute to immune evasion, which is a known feature of pancreatic cancer’s poor immunogenicity. Moreover, the relationships between USP37, TMB, and MSI suggest that USP37 may be linked with tumor heterogeneity and the mutational environment in PAAD. These findings hint at the potential utility of USP37 as a biomarker for immunotherapy responsiveness, which could be highly beneficial in pancreatic cancer. Future studies on USP37’s influence on the tumor microenvironment could provide invaluable information for designing combination therapies that enhance immune responses in PAAD.

USP37 is implicated in the initiation and progression of various tumors. For example, USP37 facilitates angiogenesis and metastasis in colorectal cancer by stabilizing β-catenin (Wu et al., 2025), while its interaction with PCNA drives osteosarcoma pathogenesis by modulating replication fork progression (Chauhan et al., 2023). However, the role and underlying mechanisms of USP37 in pancreatic cancer remain unexplored. In this study, we found that USP37 plays a significant oncogenic role in pancreatic cancer, particularly in promoting cell proliferation and tumorigenesis. The consistent overexpression of USP37 in PAAD tumors and cell lines, as well as its differential expression across various pancreatic cancer cell lines, underscores its potential as a key regulator in PC development. Our experiments demonstrated that USP37 overexpression in PANC-1 cells, which naturally express low levels of USP37, significantly enhanced cell proliferation, as evidenced by CCK-8, colony formation, EdU and RTCA assays, which further supports the notion that USP37 functions as a critical promoter of cellular proliferation in pancreatic cancer. Moreover, our *in vivo* experiments, using subcutaneous xenograft models, reinforced these findings by showing that USP37 knockdown in SW1990 cells led to significantly reduced tumor growth, whereas USP37 overexpression in PANC-1 cells resulted in enhanced tumor size and weight. These results collectively suggest that USP37 contributes to the oncogenic process by promoting both cellular proliferation and tumorigenicity in pancreatic cancer.

This study has certain limitations that should be acknowledged. First, the precise mechanisms through which USP37 exerts its effects remain unclear, requiring further investigation. Although preliminary evidence suggests that USP37 may regulate key signaling pathways associated with cell cycle control, survival, and proliferation, these pathways have not been fully elucidated. Critically, identifying specific substrates deubiquitinated by USP37 that drive pancreatic cancer pathogenesis represents a paramount focus for future investigation. Additionally, while USP37 has been identified as a potential therapeutic target due to its role in tumor growth, its therapeutic applicability in pancreatic cancer has not yet been validated in preclinical models. Future studies are needed to clarify the molecular mechanisms underlying USP37’s functions and to assess the feasibility and effectiveness of targeting USP37 for therapeutic purposes.

## 5 Conclusion

In this study, we identified that USP37 is aberrantly overexpressed in PAAD, and this overexpression is associated with poor patient prognosis. USP37 promotes PAAD progression by enhancing cell proliferation and tumorigenesis, as shown in both *in vitro* and *in vivo* models. Mechanistically, USP37 is implicated in key cancer pathways, likely through epigenetic regulation, as evidenced by DNA methylation analyses and gene set enrichment findings. Furthermore, USP37 is associated with immune regulators, TMB, and MSI, suggesting its involvement in shaping the immune microenvironment in PAAD. Collectively, our results highlight the oncogenic role of USP37 in PAAD and suggest that targeting the USP37 pathway could serve as a potential therapeutic approach to suppress tumor growth and improve immune responses in pancreatic cancer.

## Data Availability

The original contributions presented in the study are included in the article/Supplementary Material, further inquiries can be directed to the corresponding authors.

## References

[B1] Bray F. Laversanne M. Sung H. Ferlay J. Siegel R. L. Soerjomataram I. (2024). Global cancer statistics 2022: GLOBOCAN estimates of incidence and mortality worldwide for 36 cancers in 185 countries. CA Cancer J. Clin. 74, 229–263. 10.3322/caac.21834 38572751

[B2] Cao J. Wang X. Wang S. Chen Z. Tang J. (2023). Stabilization of estrogen receptor alpha by USP37 contributes to the progression of breast cancer. Cancer Sci. 114, 2041–2052. 10.1111/cas.15613 36221793 PMC10154820

[B3] Chauhan R. Gupta A. Malhotra L. Bhat A. A. Pandita R. K. Masoodi T. (2023). Ubiquitin specific peptidase 37 and PCNA interaction promotes osteosarcoma pathogenesis by modulating replication fork progression. J. Transl. Med. 21, 286. 10.1186/s12967-023-04126-2 37118828 PMC10142227

[B4] Chen B. Hu C. Jiang L. Xiang Z. Zuo Z. Lin Y. (2021). Exploring the significance of novel immune-related gene signatures in the prognosis and immune features of pancreatic adenocarcinoma. Int. Immunopharmacol. 92, 107359. 10.1016/j.intimp.2020.107359 33465729

[B5] Daassi D. Mahoney K. M. Freeman G. J. (2020). The importance of exosomal PDL1 in tumour immune evasion. Nat. Rev. Immunol. 20, 209–215. 10.1038/s41577-019-0264-y 31965064

[B6] Darling S. Fielding A. B. Sabat-Pospiech D. Prior I. A. Coulson J. M. (2017). Regulation of the cell cycle and centrosome biology by deubiquitylases. Biochem. Soc. Trans. 45, 1125–1136. 10.1042/BST20170087 28900014 PMC5652225

[B7] Dobson T. H. W. Hatcher R. J. Swaminathan J. Das C. M. Shaik S. Tao R. H. (2017). Regulation of USP37 expression by REST-associated G9a-Dependent histone methylation. Mol. Cancer Res. 15, 1073–1084. 10.1158/1541-7786.MCR-16-0424 28483947 PMC5540785

[B8] Dyba T. Randi G. Bray F. Martos C. Giusti F. Nicholson N. (2021). The European cancer burden in 2020: incidence and mortality estimates for 40 countries and 25 major cancers. Eur. J. Cancer 157, 308–347. 10.1016/j.ejca.2021.07.039 34560371 PMC8568058

[B9] Emiloju O. E. Sinicrope F. A. (2023). Neoadjuvant immune checkpoint inhibitor therapy for localized deficient mismatch repair colorectal cancer: a review. JAMA Oncol. 9, 1708–1715. 10.1001/jamaoncol.2023.3323 37676680

[B10] Frappier L. Verrijzer C. P. (2011). Gene expression control by protein deubiquitinases. Curr. Opin. Genet. Dev. 21, 207–213. 10.1016/j.gde.2011.02.005 21411309

[B11] Gu X. Zhang Q. Wu X. Fan Y. Qian J. (2021). Gene coexpression network approach to develop an immune prognostic model for pancreatic adenocarcinoma. World J. Surg. Oncol. 19, 112. 10.1186/s12957-021-02201-w 33845841 PMC8042890

[B12] He M. Zhou Z. Shah A. A. Zou H. Tao J. Chen Q. (2016). The emerging role of deubiquitinating enzymes in genomic integrity, diseases, and therapeutics. Cell Biosci. 6, 62. 10.1186/s13578-016-0127-1 28031783 PMC5168870

[B13] He M. Zhou Z. Wu G. Chen Q. Wan Y. (2017). Emerging role of DUBs in tumor metastasis and apoptosis: therapeutic implication. Pharmacol. Ther. 177, 96–107. 10.1016/j.pharmthera.2017.03.001 28279784 PMC5565705

[B14] Hernandez-Perez S. Cabrera E. Amoedo H. Rodriguez-Acebes S. Koundrioukoff S. Debatisse M. (2016). USP37 deubiquitinates Cdt1 and contributes to regulate DNA replication. Mol. Oncol. 10, 1196–1206. 10.1016/j.molonc.2016.05.008 27296872 PMC5423201

[B15] Hong K. Hu L. Liu X. Simon J. M. Ptacek T. S. Zheng X. (2020). USP37 promotes deubiquitination of HIF2α in kidney cancer. Proc. Natl. Acad. Sci. U. S. A. 117, 13023–13032. 10.1073/pnas.2002567117 32461361 PMC7293621

[B16] Kee Y. Huang T. T. (2016). Role of deubiquitinating enzymes in DNA repair. Mol. Cell Biol. 36, 524–544. 10.1128/MCB.00847-15 26644404 PMC4751696

[B17] Komander D. Clague M. J. Urbe S. (2009). Breaking the chains: structure and function of the deubiquitinases. Nat. Rev. Mol. Cell Biol. 10, 550–563. 10.1038/nrm2731 19626045

[B18] Lavin P. T. Mc Gee M. M. (2015). Cyclophilin function in Cancer; lessons from virus replication. Curr. Mol. Pharmacol. 9, 148–164. 10.2174/1874467208666150519115443 25986562

[B19] Li Y. Zhang K. Peng L. Chen L. Gao H. Chen H. (2022). Multiple perspectives reveal the role of DNA damage repair genes in the molecular classification and prognosis of pancreatic adenocarcinoma. Int. J. Mol. Sci. 23, 10231. 10.3390/ijms231810231 36142142 PMC9499455

[B20] Li S. Song Y. Wang K. Liu G. Dong X. Yang F. (2023). USP32 deubiquitinase: cellular functions, regulatory mechanisms, and potential as a cancer therapy target. Cell Death Discov. 9, 338. 10.1038/s41420-023-01629-1 37679322 PMC10485055

[B21] Li S. Yang L. Ding X. Sun H. Dong X. Yang F. (2024). USP32 facilitates non-small cell lung cancer progression *via* deubiquitinating BAG3 and activating RAF-MEK-ERK signaling pathway. Oncogenesis 13, 27. 10.1038/s41389-024-00528-z 39030175 PMC11271578

[B22] Martisova A. Holcakova J. Izadi N. Sebuyoya R. Hrstka R. Bartosik M. (2021). DNA methylation in solid tumors: functions and methods of detection. Int. J. Mol. Sci. 22, 4247. 10.3390/ijms22084247 33921911 PMC8073724

[B23] McGuire S. (2015). “World cancer report 2014. Geneva, Switzerland: world Health Organization, international agency for research on cancer, WHO press, 2015,”, 7. Geneva, Switzerland: World Health Organization, International Agency for Research on Cancer, WHO Press, 418–419. 10.3945/an.116.012211 PMC4785485 26980827

[B24] Millar A. H. Heazlewood J. L. Giglione C. Holdsworth M. J. Bachmair A. Schulze W. X. (2019). The scope, functions, and dynamics of posttranslational protein modifications. Annu. Rev. Plant Biol. 70, 119–151. 10.1146/annurev-arplant-050718-100211 30786234

[B25] Ning Z. Wang A. Liang J. Xie Y. Liu J. Feng L. (2014). USP22 promotes the G1/S phase transition by upregulating FoxM1 expression *via* beta-catenin nuclear localization and is associated with poor prognosis in stage II pancreatic ductal adenocarcinoma. Int. J. Oncol. 45, 1594–1608. 10.3892/ijo.2014.2531 24993031

[B26] Olave M. C. Graham R. P. (2022). Mismatch repair deficiency: the what, how and why it is important. Genes Chromosom. Cancer 61, 314–321. 10.1002/gcc.23015 34837268

[B27] Pan J. Deng Q. Jiang C. Wang X. Niu T. Li H. (2015). USP37 directly deubiquitinates and stabilizes c-Myc in lung cancer. Oncogene 34, 3957–3967. 10.1038/onc.2014.327 25284584

[B28] Papanicolau-Sengos A. Aldape K. (2022). DNA methylation profiling: an emerging paradigm for cancer diagnosis. Annu. Rev. Pathol. 17, 295–321. 10.1146/annurev-pathol-042220-022304 34736341

[B29] Pecina-Slaus N. Kafka A. Salamon I. Bukovac A. (2020). Mismatch repair pathway, genome stability and cancer. Front. Mol. Biosci. 7, 122. 10.3389/fmolb.2020.00122 32671096 PMC7332687

[B30] Qin T. Li B. Feng X. Fan S. Liu L. Liu D. (2018). Abnormally elevated USP37 expression in breast cancer stem cells regulates stemness, epithelial-mesenchymal transition and cisplatin sensitivity. J. Exp. Clin. Cancer Res. 37, 287. 10.1186/s13046-018-0934-9 30482232 PMC6258492

[B31] Reyes-Turcu F. E. Ventii K. H. Wilkinson K. D. (2009). Regulation and cellular roles of ubiquitin-specific deubiquitinating enzymes. Annu. Rev. Biochem. 78, 363–397. 10.1146/annurev.biochem.78.082307.091526 19489724 PMC2734102

[B32] Shen W. Song Z. Zhong X. Huang M. Shen D. Gao P. (2022). Sangerbox: a comprehensive, interaction-friendly clinical bioinformatics analysis platform. Imeta 1, e36. 10.1002/imt2.36 38868713 PMC10989974

[B33] Stromberg B. R. Singh M. Torres A. E. Burrows A. C. Pal D. Insinna C. (2021). The deubiquitinating enzyme USP37 enhances CHK1 activity to promote the cellular response to replication stress. J. Biol. Chem. 297, 101184. 10.1016/j.jbc.2021.101184 34509474 PMC8487067

[B34] Su Y. Qi R. Li L. Wang X. Li S. Zhao X. (2022). An immune-related gene prognostic risk index for pancreatic adenocarcinoma. Front. Immunol. 13, 945878. 10.3389/fimmu.2022.945878 35958614 PMC9360334

[B35] Sun Y. Yao L. Man C. Gao Z. He R. Fan Y. (2023). Development and validation of cuproptosis-related lncRNAs associated with pancreatic cancer immune microenvironment based on single-cell. Front. Immunol. 14, 1220760. 10.3389/fimmu.2023.1220760 37822927 PMC10563513

[B36] Swatek K. N. Komander D. (2016). Ubiquitin modifications. Cell Res. 26, 399–422. 10.1038/cr.2016.39 27012465 PMC4822133

[B37] Tang M. Li S. Zhu Z. Wang C. Ding S. Zhang H. (2025). USP37 counteracts HLTF to protect damaged replication forks and promote survival of BRCA1-deficient cells and PARP inhibitor resistance. Nucleic Acids Res. 53, gkaf544. 10.1093/nar/gkaf544 40548939 PMC12205991

[B39] Wang J. Gan L. Liu F. Yang Q. Deng Q. Jiang D. (2024). USP10 promotes pancreatic ductal adenocarcinoma progression by attenuating FOXC1 protein degradation to activate the WNT signaling pathway. Int. J. Biol. Sci. 20, 5343–5362. 10.7150/ijbs.92278 39430239 PMC11488585

[B40] Woo B. Baek K. H. (2019). Regulatory interplay between deubiquitinating enzymes and cytokines. Cytokine Growth Factor Rev. 48, 40–51. 10.1016/j.cytogfr.2019.06.001 31208841 PMC7108389

[B38] Wu L. Cheng C. Zhao N. Zhu L. Li H. Liu J. (2025). CDK1-mediated phosphorylation of USP37 regulates SND1 stability and promotes oncogenesis in colorectal cancer. Acta Pharm. Sin. B. 15 (4), 1938–1955. 10.1016/j.apsb.2025.02.014 40486858 PMC12138069

[B41] Wu L. Zhao N. Zhou Z. Chen J. Han S. Zhang X. (2021). PLAGL2 promotes the proliferation and migration of gastric cancer cells *via* USP37-mediated deubiquitination of Snail1. Theranostics 11, 700–714. 10.7150/thno.47800 33391500 PMC7738862

[B42] Yao L. Li J. Jiang B. Zhang Z. Li X. Ouyang X. (2023). RNF2 inhibits E-Cadherin transcription to promote hepatocellular carcinoma metastasis *via* inducing histone mono-ubiquitination. Cell Death Dis. 14, 261. 10.1038/s41419-023-05785-1 37037816 PMC10085990

[B43] Yee N. S. (2016). Immunotherapeutic approaches in pancreatic adenocarcinoma: current status and future perspectives. Curr. Mol. Pharmacol. 9, 231–241. 10.2174/1874467208666150716120810 26177643

[B44] Yu G. Wang L. G. Han Y. He Q. Y. (2012). clusterProfiler: an R package for comparing biological themes among gene clusters. OMICS 16, 284–287. 10.1089/omi.2011.0118 22455463 PMC3339379

[B45] Zhang Q. X. Wang X. C. Chen S. P. Qin X. T. (2016). Predictive value of deubiquitination enzymes USP37 in the prognosis of breast cancer. Zhonghua Yi Xue Za Zhi 96, 944–948. 10.3760/cma.j.issn.0376-2491.2016.12.008 27045719

[B46] Zhang H. He X. Yang L. Yang F. Chen R. Wen Z. (2025a). Trim45: an emerging E3 ubiquitin ligases in cancer. Cell Signal 134, 111919. 10.1016/j.cellsig.2025.111919 40466841

[B47] Zhang H. Sun F. Cao H. Yang L. Yang F. Chen R. (2025b). UBA protein family: an emerging set of E1 ubiquitin ligases in cancer-A review. Int. J. Biol. Macromol. 308, 142277. 10.1016/j.ijbiomac.2025.142277 40120894

[B48] Zhang S. Ding F. Jia F. Lu X. (2025c). USP37 as a novel regulator of NRF2 protein stability and chemoresistance in HCC. Discov. Oncol. 16, 312. 10.1007/s12672-025-01913-9 40080254 PMC11906963

[B49] Zhu J. Yang Y. Li L. Tang J. Zhang R. (2023). DNA methylation profiles in cancer: functions, therapy, and beyond. Cancer Biol. Med. 21, 111–116. 10.20892/j.issn.2095-3941.2023.0403 38062785 PMC10884540

