# Supplementary figures and images for: An integrative pan-cancer analysis of USP37 and functional validation in pancreatic cancer
Source: Front Cell Dev Biol. 2025 Aug 25;13:1659747. doi: 10.3389/fcell.2025.1659747 (PMC12415066; doi:10.3389/fcell.2025.1659747)

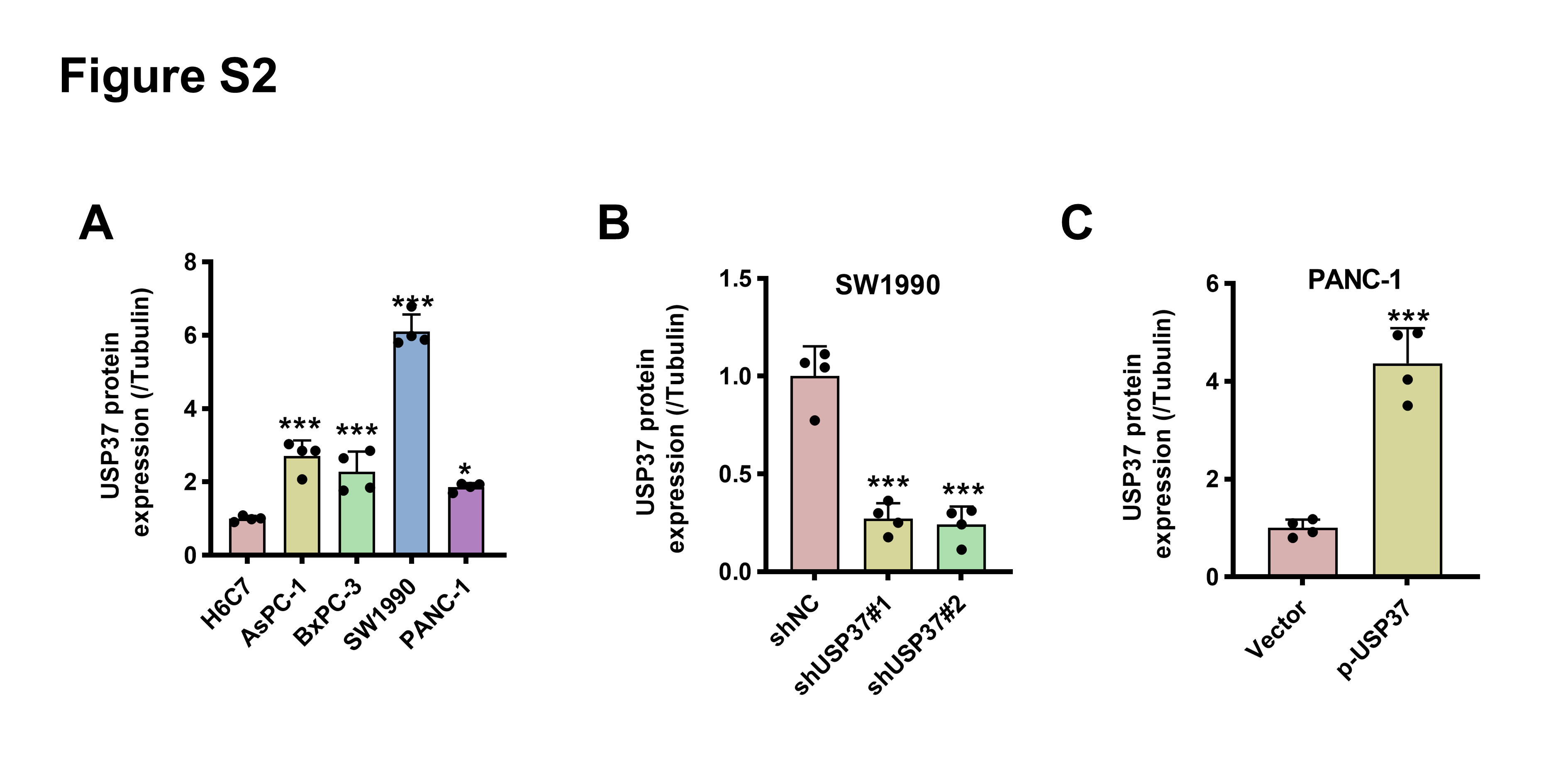

Supplement: Supplementary file 1 [file Image1.tif]
