# Supplementary material for: An integrative pan-cancer analysis of USP37 and functional validation in pancreatic cancer
Source: Front Cell Dev Biol. 2025 Aug 25;13:1659747. doi: 10.3389/fcell.2025.1659747 (PMC12415066; doi:10.3389/fcell.2025.1659747)
Supplement: Supplementary file 2 [file DataSheet1.pdf]

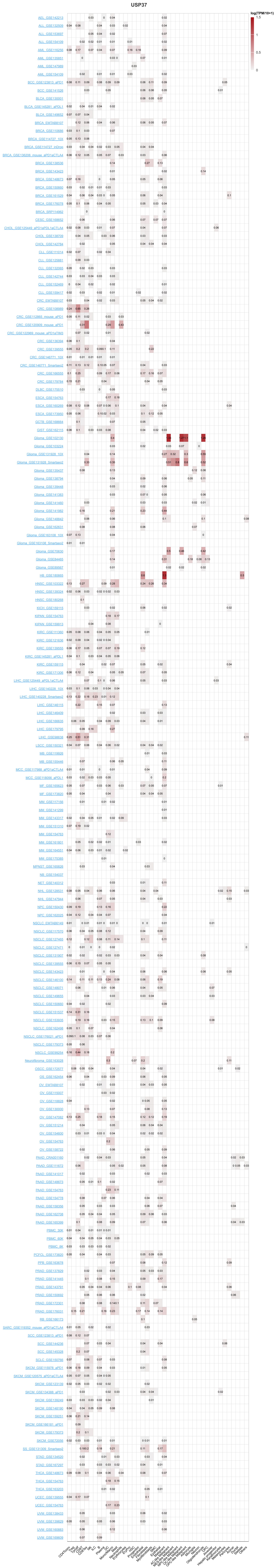
USP37
log(TPM/10+1)
1.5
1
0.5
0
